# The effectiveness of Bio-Min toothpaste in the management of white spot lesions: a randomised control trial

**DOI:** 10.1186/s13063-024-07990-5

**Published:** 2024-09-10

**Authors:** Ama Johal, Aoife Keogh, Honieh Bolooki

**Affiliations:** Centre for Oral Bioengineering, Institute of Dentistry, Queen Mary University of London, Turner Street, London, E1 2AD UK

**Keywords:** White spot lesion, Bio-Min toothpaste, Remineralisation, Fixed appliance treatment

## Abstract

**Background:**

White spot lesions (WSL) are common side effects of orthodontic treatment with fixed appliances, in which the surface layer of enamel is demineralised. Thus, remineralisation, that is a partial or complete reversal, of these lesions can occur as they affect the surface enamel. Remineralisation with low-dose fluoride, in addition to optimal oral hygiene and diet, has been recommended to manage WSL. The aim of the planned trial is to assess the effectiveness of a fluoride-containing bioactive glass toothpaste (BioMin™) in its ability to remineralise post-orthodontic demineralised WSL.

**Methods:**

A single-centre, double-blind randomised clinical trial to assess intervention with Bio-Min toothpaste on WSL forming on the teeth of young people completing orthodontic treatment.

**Discussion:**

Remineralisation of WSL can vary depending on the individual and the site of the lesion. There is a range of oral fluoride delivery methods which include toothpastes, oral rinses, and gel preparations, which can aid remineralisation of these lesions. Identifying effective methods of remineralisation to manage this common and unsightly complication of fixed appliance therapy can improve the health and aesthetics of dentition.

**Trial registration:**

ISRCTN.com International Standard Randomised Controlled Trials Number (ISRCTN) 14479893. Registered on 14 May 2020

**Supplementary Information:**

The online version contains supplementary material available at 10.1186/s13063-024-07990-5.

## Introduction

### Background and rationale {6a}

Worldwide, the demand for orthodontic treatment in young people remains high. Epidemiological evidence suggests up to two-thirds of patients demonstrate a moderate high need for orthodontic treatment [1], for which fixed appliance therapy often achieves the most optimal results [2]. Amongst the most common side effects of undertaking fixed appliance therapy is the development of white spot lesions (WSL), with a widely reported incidence of 15–85%, as a consequence of the appliance’s susceptibility to plaque accumulation, with resultant acid production and enamel demineralisation [3–5].

WSL represent surface demineralisation, rather than a sub-surface lesion with an intact surface zone, following which a degree of remineralisation takes place, with resultant partial reversal of what is an early caries lesion. Whilst the extent of remineralisation varies considerably from subject to subject and from site to site in the mouth, remineralisation of small lesions with low fluoride preparations has been recommended for lesions less than 60 mm in depth and importantly prevent arresting and consequently obtunding the lesion [6–8].

The use of fluorides, in conjunction to optimal oral hygiene and dietary advice, has been recommended to minimise this complication. As such, there is a range of methods for delivering fluoride, which include toothpaste, rinses and gel applications. A recent Cochrane review identified a clear need for further randomised control trials (RCT’s) designed to measure decalcification outcome and in doing so address the significant research design limitations identified in the current literature [9].

## Objectives {7}

Primary objective: To assess the rate and extent of remineralisation of WSL following orthodontic treatment, with a fluoride-containing bioactive glass toothpaste (BioMin™).

Secondary objective: To determine the longitudinal rate of WSL remineralisation.

Null hypothesis: A fluoride-containing bioactive glass toothpaste does not lead to a greater reduction in visible post-orthodontic WSL compared with a standard toothpaste, and post-orthodontic WSL do not reduce in size in the post-treatment period.

Primary endpoint: The average difference in percentage reduction (ADPR) of WSL will be calculated and compared between groups at the following defined points: 1 (T1), 3 (T2), 6 (T3) and 9 months (T4).

## Trial design {8}

This study is a single-centre double-blind hospital-based, randomised clinical trial, with a parallel design, assessing the rate and extent of remineralisation of WSL following orthodontic treatment, with a fluoride-containing bioactive glass toothpaste (BioMin™).

Ethical approval was obtained from the Wales Research Ethics Committee 6 Swansea (REC ref: 21/WA/0256). Written informed consent will be obtained from those participants meeting the selection criteria and agreeing to partake in the study, following the provision of a patient information leaflet.

Participants will be randomly assigned into either Intervention care (IC) or Standardised care (SC) and both the operator and participant will be blinded to treatment assignment. Participants will be provided with either a fluoride-containing bioactive glass toothpaste (BioMin™) or a standard fluoride-containing toothpaste (Colgate®) and instructed to brush their teeth with the toothpaste twice a day using a standard manual toothbrush (Oral B Indicator Flat Trimmed Manual Toothbrush).

Baseline demographics and high-quality intra-oral photographs of the teeth will be obtained in line with routine start and end of treatment records. Intra-oral images will be obtained (T0), within 7 days of appliance removal (debond), in a standardised and reproducible manner, in a darkened room using a polarising lens [10]. Quantitative light-induced fluorescence therapy will also be utilised. A maximum of 4 WSL will be selected from each participant, representing a range of different teeth, with each lesion measured independently. Plaque scores, measured using the Turesky modification of the Quigley–Hein plaque index [11], and gingival health, measured using the Loe and Silness gingival index [12], will also be recorded at all time points (T0-T4).

## Methods: participants, interventions and outcomes

### Study setting {9}

The study will be undertaken at the Royal London Dental Hospital, Bart’s Health NHS Trust in line with the CONSORT guidelines.

### Eligibility criteria {10}

Inclusion criteria:

- Young people (aged 15–21 years)
- Undergone a minimum of 12 months of fixed appliance treatment in both arches, with clinically identifiable WSL present on at least 1 of the maxillary and/or mandibular anterior teeth (first premolar to first premolar) at the time of removal of fixed orthodontic appliances
- Optimal dental health and oral hygiene prior to treatment

Exclusion criteria:

- Participants who have undergone previous courses of fixed appliance treatment
- Pre-existing clinical evidence of either enamel structural defects, demineralised lesions or fluorosis prior to commencement of treatment

### Who will take informed consent? {26a}

Participants aged 16 years and older will be provided with a patient information leaflet regarding the study. For participants aged under 16 years, the parents/guardians will be provided with a parent/guardian information sheet regarding the study.

Informed consent would be obtained by the investigators (AJ, AK or HB).

Participants aged 16 years and older who agree to partake in the study will provide informed written consent via a consent form. For participants aged under 16 years, the parents/guardians will provide informed written consent via a consent form.

Participants would be re-consented if they turned 16 years of age during the study.

### Additional consent provisions for collection and use of participant data and biological specimens {26b}

N/A—there are no ancillary studies planned.

### Interventions

#### Explanation for the choice of comparators {6b}

Participants will be randomised to receive either the fluoride-containing bioactive glass toothpaste (BioMin™) or a standard fluoride-containing toothpaste (Colgate®). BioMin™ toothpaste contains 600-ppm fluoride, whereas the standard toothpaste (Colgate® Total, Colgate Palmolive, USA) contains 1450-ppm fluoride. Both will be presented in a non-proprietary labeled and similar format, to maintain blinding.

#### Intervention description {11a}

Participants randomised to the IC group will be issued with a 3-month supply of BioMin™ toothpaste, each being labelled and packaged to appear exactly the same as the standard fluoride-containing toothpaste in the SC group, with a unique identifier number label generated from the randomisation sequence. Each participant will be instructed to clean their teeth using a standard manual toothbrush (Oral B Indicator Flat Trimmed Manual Toothbrush) and a daily regime of brushing with the allocated toothpaste twice daily.

#### Criteria for discontinuing or modifying allocated interventions {11b}

N/A—we do not see any specific criteria for discontinuing or modifying allocated interventions as we will be carrying out routine clinical care using commercially available toothpastes.

#### Strategies to improve adherence to interventions {11c}

Participants will be reminded and encouraged to use the allocated trial toothpaste, during the regular routine planned follow-up appointments, after their appliances have been removed.

#### Relevant concomitant care permitted or prohibited during the trial {11d}

No relevant concomitant care is prohibited during the trial.

#### Provisions for post-trial care {30}

Participants will be reviewed in the hospital for at least 12 months post-debond of fixed appliances. Following this, patients may be discharged into the care of their general dental practitioner for their ongoing dental care, in line with the established routine clinical practice.

### Outcomes {12}

Primary outcome: Rate and extent of remineralisation of WSL following orthodontic treatment, with a fluoride-containing bioactive glass toothpaste (BioMin™) will be assessed during the follow-up period and reported in terms of the final trial end-point of 9 months (T4).

Secondary outcome: Longitudinal rate of WSL remineralisation.

### Participant timeline {13}

The participant timeline is found in Table 1.

**Table 1:** Schedule of assessment

Assessment	Baseline T0	1 month T1	3 months T2	6 months T3	9 months T4
Intra-oral clinical photographs	X	X	X	X	X
Plaque scores	X	X	X	X	X
Gingival health	X	X	X	X	X
Age	X				
Gender	X				

**Fig. 1:**
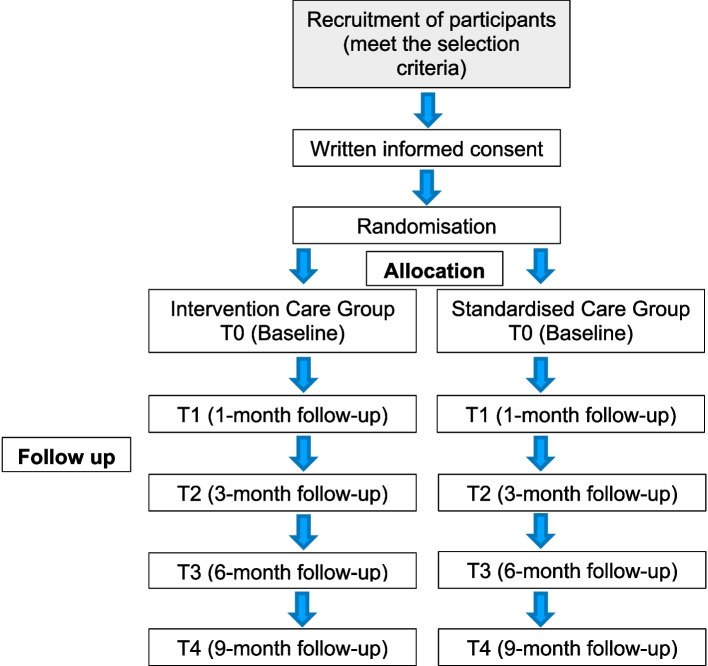
Study scheme diagram

The study scheme diagram is found in Fig. 1.

### Sample size {14}

Estimation of the sample size was based on establishing that a clinically meaningful difference between the groups and this would be at least 20% in enamel surface defect. Expected standard deviations were based on previous studies, which were about 15% [8]. Otherwise, we assume a significance level of 0.05 and 0.90 for power. A sample size determination was obtained in G*power 3.1 software [13], using the *t* test for independent samples platform. Under this circumstance, the minimal sample size should be 26 participants (13 in each group). Assuming a 20% dropout rate (5.2 participants), the total sample size should be 32, equally distributed between the groups.

**Fig. 2:**
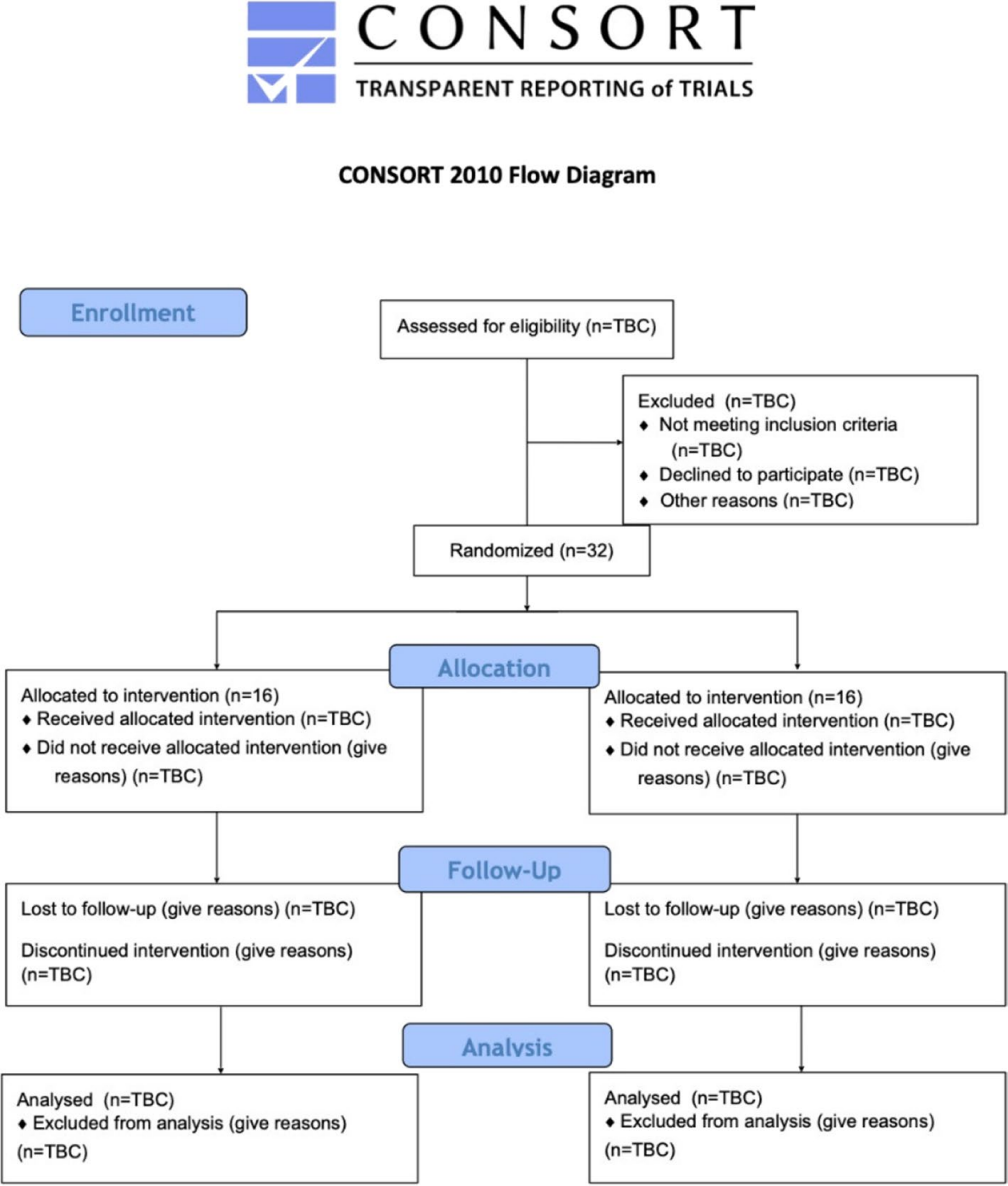
CONSORT flow diagram

The CONSORT flow diagram is found in Fig. 2.

### Recruitment {15}

Participants will be recruited from patients who have had orthodontic treatment carried out at the Royal London Dental Hospital, Bart’s Health NHS Trust. Participants who meet the eligibility criteria will be recruited by the investigators (AJ, AK and HB) at debond appointments or retainer fit appointments following the debonding of fixed appliances.

## Assignment of interventions: allocation

### Sequence generation {16a}

A simple computer-generated randomisation method will be performed using a random number sequence (www.graphpad.com/quickcalcs/randomn2.cfm) to ensure the equivalence of numbers in each group.

### Concealment mechanism {16b}

Randomisation sequences will be placed in opaque sealed envelopes to ensure allocation concealment.

### Implementation {16c}

The chief investigator (AJ) will generate the allocation sequence and assign participants to the interventions. Participant enrolment will be carried out by the investigators (AK and HB).

## Assignment of interventions: blinding

### Who will be blinded {17a}

Each participant will be anonymised, and both the operator and participant blinded to treatment assignment.

### Procedure for unblinding if needed {17b}

N/A—it is not anticipated that unblinding would be required.

## Data collection and management

### Plans for assessment and collection of outcomes {18a}

- Questionnaire responses
- Intra-oral clinical photographs of the teeth
- Plaque scores [11]
- Gingival index [12]

The intra-oral clinical photographs, plaque scores and gingival index will be carried out by the same trained and calibrated assessors, within the research team.

### Plans to promote participant retention and complete follow-up {18b}

The participants will be reminded of the aesthetic benefits of applying the toothpaste to minimise the detrimental harm to the enamel and improve the overall appearance and we hope this will promote both their retention and likelihood to complete the follow-up.

### Data management {19}

The data recorded will be stored securely both physically and electronically. The data will be accessed at Queen Mary University of London by the CI and research student only and will be password protected. The personal data of the participants will be securely held within the research facilities at the Queen Mary University of London, by the chief investigator. Data transfer for analysis will be undertaken in an anonymised form, using unique ID numbers and password-protected access.

### Confidentiality {27}

Information related to participants will be kept confidential and managed in accordance with the Data Protection Act 2018, NHS Caldecott Principles, The Research Governance Framework for Health and Social Care, and the conditions of Research Ethics Committee Approval.

### Plans for collection, laboratory evaluation and storage of biological specimens for genetic or molecular analysis in this trial/future use {33}

N/A—biological specimens have not been planned for collection.

## Statistical methods

### Statistical methods for primary and secondary outcomes {20a}

An intention-to-treat analysis will be applied, with the Statistical Package for Social Sciences (SPSS Inc., Version 24, New York, USA) used for data analysis, with means (SD) or medians and inter-quartile ranges.

- To assess the aims of the study, we will test each of the repeated measures first by themselves using a repeated measures ANOVA controlling for groups.
- Data will be collected at 1 (T1), 3 (T2), 6 (T3) and 9 (T4) months and analysed to assess the effectiveness of intervention care in comparison with the standardised care.

To make meaningful comparisons in decalcification between groups, the method described by Willmot et al. [10] will be used to calculate the average WSL score per subject. We can then compare that figure for the whole subject with other participants in the clinical trial in order to compare test and control groups. Thus, the difference in WSL, as a proportion of total tooth area (DWSL%t) for A–B (where A represents baseline and B follow-up), will be expressed as a percentage reduction (DPR). For participants with multiple lesions, a maximum of 4 WSL will be measured and averaged to give the average difference in percentage reduction (ADPR), whereas for participants with only a single WSL, a single measurement will be used. The average difference in percentage reduction (ADPR) will then be calculated and compared between groups at defined points (1, 3, 6 and 9 months). The following measurements will be calculated:

DWSL%t: The demineralised white spot lesion as a percentage area of the visible labial surface of the tooth calculated by:

$$\mathrm{DWL\%t}=\frac{\mathrm{Area\ of\ white\ spot\ lesion\ }({\text{WSL}}) \times 100}{\mathrm{Area\ of\ labial\ surface\ of\ tooth\ }(t)}$$

DPR(x): The difference in percentage reduction at *x* months for each tooth. The difference of DWSL%t for A–B, where A represents baseline and B follow-up at *x* (1, 3, 6 and 9 months).

ADPR(x): The average difference in percentage reduction for each subject between debond and* x* months. Calculated from the mean of XY plots for each tooth against time of DWL%t. This will be calculated for 1, 3, 6 and 9 months for each subject.

### Interim analyses {21b}

The chief investigator (CI) will make the final decision to terminate the trial.

### Methods for additional analyses (e.g. subgroup analyses) {20b}

N/A—no additional analyses planned.

### Methods in analysis to handle protocol non-adherence and any statistical methods to handle missing data {20c}

All randomised participants will be included in the main analyses and an intention-to-treat analysis will be applied.

### Plans to give access to the full protocol, participant-level data and statistical code {31c}

N/A—no plans to give access.

## Oversight and monitoring

### Composition of the coordinating centre and trial steering committee {5d}

The trial is being led by the CI (AJ) and is being carried out by two investigators (AK and HB).

### Composition of the data monitoring committee, its role and reporting structure {21a}

The data recorded in this study will be stored securely both physically and electronically. The data will be analysed in the Queen Mary, University of London, by the CI (AJ) or the investigators (AK, HB). The data will be password protected and only the CI and the investigators will have full access. The personal data of the participants will be securely held within the research facilities at the Queen Mary, University of London, by the CI.

### Adverse event reporting and harms {22}

We expect no adverse events in the study, as it is questionnaire-based and relates to routine clinical care, with the use of commercially available toothpastes.

### Frequency and plans for auditing trial conduct {23}

The sponsor or delegate retains the right to audit any study, study site or central facility. In addition, the funders may audit any part of the study where applicable.

### Plans for communicating important protocol amendments to relevant parties (e.g. trial participants, ethical committees) {25}

Any protocol amendments will be communicated to the sponsor (Joint Research Management Office), the relevant research ethics committee and the study participants.

## Dissemination plans {31a}

It is intended that the results of the research would be presented at conferences/attending workshops. In addition, the results will be published in peer-reviewed journal.

## Discussion

N/A—all issues discussed in previous sections.

## Trial status

Protocol version 02, Dated: 25/08/2021.

Date recruitment began: 07/06/2023.

Approximate date of recruitment completion: 01/12/2024.

## Supplementary Information

**Supplementary Material 1. **

**Supplementary Material 2. **

## Data Availability

The final dataset will be accessible by the CI (AJ) and the investigators (AK and HB).

## Abbreviations

ADPR: Average difference in percentage reduction
CI: Chief investigator
DPR: Difference in percentage reduction
DWSL: Difference in white spot lesion
IC: Intervention care
SC: Standardised care
T0: Baseline
T1: 1-Month follow-up observation
T2: 3-Month follow-up observation
T3: 6-Month follow-up observation
T4: 9-Month follow-up observation
WSL: White spot lesions
